# Malacological survey and geographical distribution of vector snails for schistosomiasis within informal settlements of Kisumu City, western Kenya

**DOI:** 10.1186/1756-3305-4-226

**Published:** 2011-12-07

**Authors:** Selpha Opisa, Maurice R Odiere, Walter GZO Jura, Diana MS Karanja, Pauline NM Mwinzi

**Affiliations:** 1 Neglected Tropical Diseases Branch, Centre for Global Health Research, Kenya Medical Research Institute, P. O. Box 1578-40100, Kisumu, Kenya; 2 Department of Zoology, Maseno University, P. O. Box 333-40105, Maseno, Kenya

**Keywords:** *Biomphalaria*, *Bulinus*, malacology, schistosomiasis, snails, informal settlement, urban area

## Abstract

**Background:**

Although schistosomiasis is generally considered a rural phenomenon, infections have been reported within urban settings. Based on observations of high prevalence of *Schistosoma mansoni *infection in schools within the informal settlements of Kisumu City, a follow-up malacological survey incorporating 81 sites within 6 informal settlements of the City was conducted to determine the presence of intermediate host snails and ascertain whether active transmission was occurring within these areas.

**Methods:**

Surveyed sites were mapped using a geographical information system. Cercaria shedding was determined from snails and species of snails identified based on shell morphology. Vegetation cover and presence of algal mass at the sites was recorded, and the physico-chemical characteristics of the water including pH and temperature were determined using a pH meter with a glass electrode and a temperature probe.

**Results:**

Out of 1,059 snails collected, 407 (38.4%) were putatively identified as *Biomphalaria sudanica*, 425 (40.1%) as *Biomphalaria pfeifferi *and 227 (21.5%) as *Bulinus globosus*. The spatial distribution of snails was clustered, with few sites accounting for most of the snails. The highest snail abundance was recorded in Nyamasaria (543 snails) followed by Nyalenda B (313 snails). As expected, the mean snail abundance was higher along the lakeshore (18 ± 12 snails) compared to inland sites (dams, rivers and springs) (11 ± 32 snails) (F_1, 79 _= 38.8, P < 0.0001). Overall, 19 (1.8%) of the snails collected shed schistosome cercariae. Interestingly, the proportion of infected *Biomphalaria *snails was higher in the inland (2.7%) compared to the lakeshore sites (0.3%) (P = 0.0109). *B. sudanica *was more abundant in sites along the lakeshore whereas *B. pfeifferi *and *B. globosus *were more abundant in the inland sites. *Biomphalaria *and *Bulinus *snails were found at 16 and 11 out of the 56 inland sites, respectively.

**Conclusions:**

The high abundance of *Biomphalaria *and *Bulinus *spp. as well as observation of field-caught snails shedding cercariae confirmed that besides Lake Victoria, the local risk for schistosomiasis transmission exists within the informal settlements of Kisumu City. Prospective control interventions in these areas need to incorporate focal snail control to complement chemotherapy in reducing transmission.

## Background

*Schistosoma mansoni *infection continues to be one of the most important and widespread of the neglected tropical diseases in Kenya, especially among communities living around the shores of Lake Victoria in western Kenya [1,2]. The distribution of the disease is determined, to a large extent, by the presence or absence of *Biomphalaria *snails, which act as the parasite's intermediate host. The majority of research on schistosomiasis lays emphasis on disease prevalence and intensity of infection among human populations, occasionally trying to identify the intermediate snail hosts within the vicinity of the areas. Few malacological surveys have been carried around Lake Victoria [3,4] and around Kisumu City [5]. Such surveys not only serve to assess local transmission, but also help to elucidate associations between disease point prevalences, which in turn, aid in creating more accurate predictive maps of expected schistosome distributions.

The World Health Organization (WHO) has emphasized the need to create predictive maps for expected schistosome distributions. In this regard, the Geographical Information System (GIS) can be applied to consider the spatial patterns of intermediate host snails simultaneously with those of human infection so as to improve efficiency of allocation for available transmission control interventions. Recent malacology surveys around Lake Victoria [3,6,7] provide an insight on the usefulness of integrating snail surveillance with parasitology data from humans.

However, several knowledge gaps still exist in integrating snail distribution with human infection data. First, fragmentation of infection among human populations versus snail sampling makes it difficult to indicate with certainty the occurrence and distribution of the schistosome snail host in the areas. This is further confounded by the fact that most human populations within endemic areas exhibit high itinerancy [3], thus complicating the pattern for locally acquired versus imported infections. Second, although chemotherapy plays a significant role in reducing morbidity and mortality due to schistosomiasis, the costs and logistical constraints hamper its effectiveness on a wider scale. Parallel preventive measures such as snail control, whose integration requires a thorough understanding of snail distribution, therefore, seem plausible. Third, the longevity of schistosome infections in the human host makes it difficult to detect when and where transmission actually occurs, without undertaking snail surveillance. Since snails are obligatory hosts for the larval stages of schistosomes, their examination provides important information on active transmission foci. Both the parasite and the vector must be targeted in order to break the cycle of transmission so as to achieve success in controlling schistosomiasis.

The objective of this study was to determine the presence and geographical distribution of *Biomphalaria *and *Bulinus *snails, and their infection prevalence among freshwater habitats in informal urban settlements and along the lakeshore in Kisumu City, western Kenya. In addition, we also assessed the environmental and physicochemical factors that may influence snail distribution. This was a follow-up corroborative study to our previous work on schistosomiasis among school children in these informal settlement areas, where we reported a prevalence of 21% and 3.6% for *S. mansoni *and *S. haematobium*, respectively [8].

## Methods

### Study area and water contact activities

The study was conducted in Kisumu City, which borders Lake Victoria in western Kenya, in April-May 2011. Kisumu is the third largest urban centre in Kenya with an area of 417 sq. km (157 sq. km. of water and 260 sq. km. of land) and a population estimated at 500,000 [9]. The urban area consists of eight informal settlement areas, namely Bandani, Kaloleni, Manyatta A, Manyatta B, Nyalenda A, Nyalenda B, Nyamasaria and Obunga [9]. In the Lake Victoria region, the lake is the primary source of *S. mansoni *infection, with an inverse association between distance to the lake and prevalence of infection [2]. Rainfall pattern in Kisumu is seasonally bimodal, with the heaviest rains falling from March through May and the shorter rainy season occurring in November and December. A perennial river (River Nyamasaria) and seasonal streams (Auji and Wigwa) flow through the area. There are also 4 dams (Kanyamedha, Molem, Kasule and Whitestone).

### Water bodies and water-contact activities

**Figure 1:**
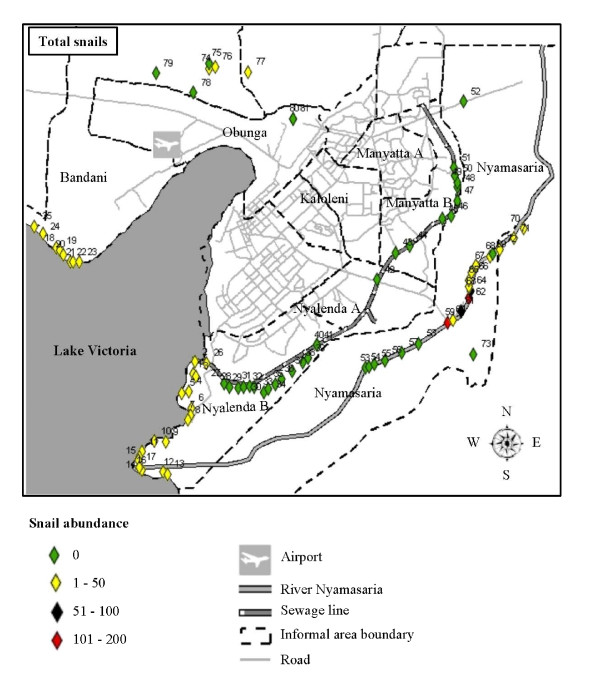
**Malacological results of 81 sites surveyed for *Biomphalaria *and *Bulinus *snails within the informal settlement areas of Kisumu City, Kenya, April-May 2011**. **Sites are coloured by abundance**.

Survey sites were selected, on the basis of preliminary field observations as obvious water contact points where people consistently go to collect water, wash clothes, bathe, swim or play (young children) and where there was car washing and sand harvesting activities. Briefly, sites 1-25 are along the Lakeshore, sites 26-41 along River Wigwa, sites 42-51 along River Auji, site 52 at Suedoy dam, sites 53-72 along River Nyamasaria, site 73 at Molem dam, sites 74-76 at Kanyamedha dam, site 77 at Whitestone dam, and sites 78-81 are on springs (Figure 1).

### Snail sampling

Snail sampling was conducted in April-May 2011, in sites where there was major human water contact within 6 informal settlements (Bandani, Manyatta B, Nyamasaria, Nyalenda A, Nyalenda B and Obunga) and along the shores of Lake Victoria. Two informal areas (Kaloleni and Manyatta A) were excluded from the survey since the main sources of water were kiosks selling piped water and mobile water vendors. Inland habitat sampling sites (within the informal settlements) included, dams, rivers and springs). Sampling was carried out by 2 trained field collectors using standard snail scoops or occasionally, by hand collection. The same collectors scooped for snails throughout so as to achieve some level of standardized sampling effort. Sampling time was fixed at 30 minutes per location and was performed between 08:30 h and 10:30 h. Sampling area per location was approximately 5 m^2^, whereas lengths of 10 metres along streams and lake shoreline were used. At each collection time, snails from each site were appropriately labelled and transported in separate perforated plastic containers to the Ministry of Health's Division of Vector-Borne and Neglected Tropical Diseases (DVBNTD) laboratory, Kisumu, where they were processed. By 11:00 h, snails were rinsed and placed individually in 24-well culture plates containing 1 ml of clear, filtered water (same source as site of collection) and exposed to indirect sunlight for 4 h to induce cercarial shedding. The time of cercariae shedding was carefully selected to coincide with the early peak shedding time (midday) [4]. The wells of the plates were then examined for the presence of cercariae under a dissecting microscope. Snails that did not shed cercariae on the first exposure were re-exposed on the second day. Bifurcate cercariae were used to indicate that the cercariae were of mammalian origin. Snails were identified to species level based on shell morphological characteristics using standard keys [10,11]. Snails were killed and stored in 70% alcohol for prospective analysis and confirmation of species using molecular techniques.

### Geographical distribution of snails

To determine the geographic distribution of snails, all sampled habitats were mapped using hand-held differential geographic global positioning system (GPS) units (Trimble Navigation Ltd, California, USA) with an estimated accuracy of ± 1 metre [12]. Data were downloaded with differential correction into a GPS database (GPS pathfinder office 2.8 Trimble Navigation Ltd, California, USA) and analyses performed using ArcView version 9.2 software (Environmental Systems Research Institute, Inc., Redlands, CA). We determined the abundance of *Biomphalaria *and *Bulinus *snails, categorized as 0, 1-50, 51-100, 101-200.

### Environmental and physico-chemical characteristics

We chose environmental and physico-chemical variables suggested to be important for freshwater snail distributions. In this regard, we recorded vegetation cover and presence of algal mass. The physico-chemical characteristics of the water at each sampling site including pH and temperature were determined using a pH meter with a glass electrode and a temperature probe (CyberScan pH310, model # ***WAG-WE30220, Wagtech WTD***, Palintest Ltd, Gateshead, UK).

### Data analysis

All analyses were performed using statistical analysis software (SAS) (v. 9.2; SAS Institute Inc., Cary, NC, USA) and *P *values < 0.05 were considered statistically significant. Unless otherwise indicated, values are presented as means ± S.D. Data were checked for normality and homogeneity of variance and log-transformed [log (x + 1)] when necessary, but only non-transformed means are reported. A one-way analysis of variance (ANOVA) was used to compare the difference in snail abundance between the sites along the lakeshore and those in inland habitats. Post-hoc Bonferroni adjustment (where appropriate) was used to account for multiple comparisons. Comparisons for prevalence of infection between *Biomphalaria *and *Bulinus *spp. and prevalence of infection for *Biomphalaria *spp. between the lakeshore and inland sites were performed using Fisher's exact test. Associations between snail abundance and environmental/physico-chemical variables were determined using spearman correlations (r_s_).

## Results

### Snail species, distribution and abundance

A total of 1,059 freshwater snail specimens were collected from 81 different sampling sites, 25 along the shores of Lake Victoria and 56 from inland habitats (dams, rivers and springs) within the informal settlements. On the basis of shell morphology, 407 (38.4%) of the snails collected were putatively identified as *Biomphalaria sudanica*, 425 (40.1%) as *Biomphalaria pfeifferi*, whereas 227 (21.5%) were identified as *Bulinus globosus *(Table 1). Of the *Biomphalaria *spp., 48.9% belonged to *B. sudanica *and 51.1% were *B. pfeifferi*. Other than *Biomphalaria *and *Bulinus *species (the intermediate host snails of *S. mansoni *and *S. haematobium*, respectively), the other commonly identified freshwater snail was *Lymnaea natalensis*.

**Table 1:** Summary of the distribution of snails collected among the 6 informal settlement areas of Kisumu City, Kenya, April -May 2011

Informal area	Number of sites	Snail species			Total snail abundance	Mean snail abundance^1^
		*B. sudanica *	*B. pfeifferi*	*B. globosus*		
Bandani	14	107	33	63	203	15 ± 14
Manyatta B	10	0	0	0	0	0
Nyalenda A	6	0	0	0	0	0
Nyalenda B	27	159	63	91	313	12 ± 13
Nyamasaria	22	141	329	73	543	25 ± 47
Obunga	2	0	0	0	0	0

^1^Values are means ± SD (rounded off to whole numbers).

The spatial distribution of snails was clustered, with few sites accounting for most of the snails. As expected, all the 25 sites sampled along the lakeshore yielded snails, with 17 sites (68%) yielding 11-50 snails (Figure 1). Overall, of the 81 sites surveyed, 39 (all inland sites) did not yield any snail (Figure 1), but notably, the 4 sites with the highest snail densities (> 51 snails) were inland sites (Figure 1). *Biomphalaria *and *Bulinus *snails were found at 16 and 11 out of the 56 inland sites, respectively. Nyalenda B recorded the highest number of sites with 1-50 snails (18 sites), whereas only Nyamasaria had sites with 51-100 snails (2 sites) and 101-200 snails (2 sites) (Figure 1). Sampled sites within Manyatta B, Nyalenda A and Obunga informal areas did not yield any snail.

The log-transformed mean snail abundance varied significantly across the 6 informal areas (F_5, 75 _= 4.93, P = 0.0006), with Nyamasaria recording the highest abundance (Table 1). Similarly, mean snail abundance was different based on the type of water body sampled (F_3, 77 _= 13.63, P < 0.0001), with the Lakeshore recording the highest mean abundance (Table 2). Mean snail abundance at the lakeshore was higher compared to rivers (P < 0.0001), but was marginally higher compared to dams (P = 0.0502) (Table 2). Springs did not yield any snails. We also compared the snail abundance at the lakeshore sites versus the inland sites (pooled by site). As expected, the log-transformed mean snail abundance was higher along the Lakeshore (18 ± 12 snails) compared to inland sites (11 ± 32 snails) (F_1, 79 _= 38.8, P < 0.0001).

**Table 2:** Distribution of snails among water bodies in Kisumu City, Kenya, April -May 2011

Site type	Number of sites	Snail species			Total snail abundance	Mean snail abundance^1^
		*B. sudanica *	*B. pfeifferi*	*B. globosus*		
Dams	6	1	9	39	49	8 ± 16^b^
Lakeshore	25	261	80	112	453	18 ± 12^a^
Rivers	46	145	336	76	557	12 ± 34^b^
Springs	4	0	0	0	0	0

^1^Different lowercase letters indicate significant difference for site type (P < 0.05). Values are means ± SD (rounded off to whole numbers).

**Figure 2:**
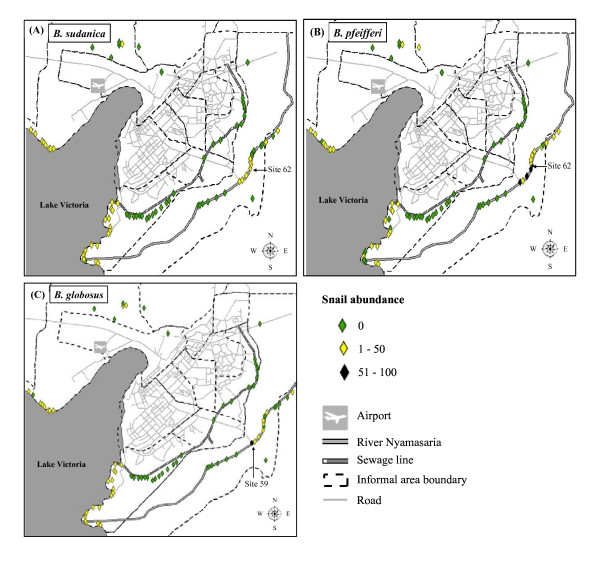
**Spatial distribution of snails by species abundance; (A) *Biomphalaria sudanica*, (B) *Biomphalaria pfeifferi*, (C) *Bulinus globosus *within the informal settlement areas of Kisumu City, Kenya, April-May 2011**. **Sites are coloured by abundance**.

Distribution of *Biomphalaria *spp. revealed that *B. sudanica *routinely occurred along the lakeshore (76.5%) while *B. pfeifferi *was dominant at the inland sites (70%) (Table 2; Figures 2A &2B). The lakeshore and inland sites had a fairly similar abundance of *B. globosus *(Table 2). The maximum number of *B. sudanica *and *B. pfeifferi *collected from a single location was 49 and 98 snails, respectively, both collected at site 62 (along River Nyamasaria) (Figures 2A &2B). On the other hand, the maximum number of *B. globosus *collected from a single location was 58 snails, at site 59 (along River Nyamasaria) (Figure 2C).

### Infection in snails

Surprisingly, very few of the snails collected during the survey were found to be shedding cercariae. Overall, regardless of species, 19 (1.8%) of the snails were infected. The natural prevalence of shedding schistosome cercariae was 1.7% in *B. sudanica*, 1.6% in *B. pfeifferi *and 2.2% in *B. globosus*. The infection prevalence was higher, but not significantly different in *Bulinus *spp. (2.2%) compared to *Biomphalaria *spp. (1.7%). Rather surprisingly, of the *Biomphalaria *snails that shed cercariae, 13 were from the inland sites while only 1 was from the Lakeshore site. The proportion of infected *Biomphalaria *snails was higher in the inland sites (2.7%) than the Lakeshore sites (0.3%) (P = 0.0109, by Fisher's exact test). *Biomphalaria *snails were found shedding *S. mansoni *cercariae at 5 sites (site 5 at lakeshore and sites 59, 61, 64 and 65 along River Nyamasaria). On the other hand, 5 *Bulinus *snails from inland sites shed cercariae, and interestingly, all these snails were collected from River Nyamasaria and Kanyamedha dam. *Bulinus *snails were found shedding cercariae at only 2 sites (site 59 along River Nyamasaria and site 76 at Kanyamedha dam). Infected *Bulinus *snails were absent from the lakeshore sites and so comparison for proportion of infected snails between inland and lakeshore sites was not possible.

### Environmental and physico-chemical factors

The most common vegetation covers identified were floating macrophytes i.e. water hyacinth (*Eichhornia crassipes*) and water lily (*Nymphaea *spp.). However, vegetation cover was not associated with snail abundance.

The mean water temperature at the lakeshore sites (29.4 ± 1.4°C; range = 27.4-32.4°C) was higher than that at the inland sites (26.4 ± 1.9 °C; range = 22.5-33°C) (F_1,79 _= 49.47, P < 0.0001). The pH levels of water varied greatly at the sites. The mean pH at the lakeshore sites was 8.7 ± 1.1 (range = 6.7-11.2) and 8.5 ± 1.2 (range = 5.9-11.1) at the inland sites, but the difference was not significant. There was a positive association between water temperature and overall snail abundance (r = 0.3, n = 81, P = 0.0195). Neither vegetation cover nor pH was associated with snail abundance.

## Discussion

This study has been novel in attempting to elucidate the distribution of schistosome snail species in an urban setting. Examination of over 1,059 snails showed that *B. sudanica *and *B. pfeifferi *were the most abundant snails, ~ 2 times more common than *B. globosus*, but the natural prevalence of shedding schistosome cercariae was similar between *Biomphalaria *and *Bulinus *spp. The spatial distribution of snails was clustered, with few sites accounting for most of the snails. *B. sudanica *and *B. pfeifferi *snails were more abundant along the lakeshore and inland sites, respectively.

### Snail abundance and distribution

Lake Victoria has been known to be the main source of transmission for intestinal schistosomiasis in western Kenya [2]. However, the high abundance of *Biomphalaria *and *Bulinus *spp. as well as field-caught snails shedding cercariae, together with our recent findings of high prevalence of the intestinal schistosomiasis [8], confirms that besides the Lake, there is local risk-of schistosomiasis infection within Kisumu City. Interestingly, all the *Biomphalaria *snails from the inland sites that shed cercariae came from 4 sites along River Nyamasaria, which flows adjacent to Rae Kanyaika primary school that had one of the highest *S. mansoni *prevalence (61%) in our previous survey [8]. Similarly, high snail abundance and presence of infected *Bulinus *snails (2.2%) along River Nyamasaria (in Nyamasaria) and around Kanyamedha dam (in Bandani) are in congruence with our survey among school children [8] that showed *S. haematobium *prevalence of 3.5% and 6.8% for the two areas, respectively, and suggests that these areas are local hotspots for urogenital schistosomiasis. Our data revealed a geographic stratification for species within the City. Whereas *B. sudanica *was abundant in sites along lakeshore, *B. pfeifferi *was abundant in inland sites, preferring smaller, man-made habitats such as dams and rivers, consistent with previous research [13]. *B. globosus *snails were also abundant in the inland sites. *B. pfeifferi *and *B. sudanica *are known to prefer shallow/swampy water, with plant detritus as a substratum [14], whereas *B. globosus *prefers shallow water, where it may occur on bare substrata, but commonly among aquatic plants [15]. Preference for different environmental conditions such as abundant microflora, depth of water, oxygen content and other physico-chemical factors and natural behavioral mode of adaptation may explain why the snail species showed marked differences in each locality.

### Environmental and physico-chemical factors on snail abundance and distribution

Environmental factors that influence snail distribution are often overlooked, despite the fact that these can vary considerably from site to site and area to area, even within short distances [16]. Among the physico-chemical variables measured in this study, water temperature appeared to be the key determinant of snail abundance. The positive association between snail abundance and water temperature observed in our study is in agreement with observations from Uganda that, snail distributions were restricted in the north and north-eastern parts of the country with high temperatures [7]. In addition, it has been demonstrated that *B. pfeifferi *grew and survived better at 25°C than at 19°C [17]. However, in contrast to our findings, Kariuki and others [18] did not find any association between snail abundance and water temperature, and suggested that this may have been due to the narrow range of temperature in their study. In the same study, vegetation type was significantly associated with presence of several snail species. The present study scored for presence or absence of vegetation cover and not by type.

Of noteworthy was how widely the pH values varied; snails were found in sites with pH ranging from 6.7 to more than 11. It has been suggested that such high pH values may be caused by human contaminants such as cleaning products or may be attributable to the acquisition of H+ ions as occurs during photosynthesis at daylight hours [19]. The absence of association between pH and snail abundance observed in our study has also been reported previously [5] and suggests that pH may not be a key determinant of snail abundance, as is the case with other freshwater organisms [20]. However, on the contrary, Levitz and others [21] have reported that a lower pH (more acidic) was associated with higher snail abundance. This discordance in findings on the association between pH and snail abundance remains to be elucidated.

### Cercarial shedding and implications for transmission

A very low proportion of snails in our study shed cercariae. *S. mansoni *cercariae are diurnal and are typically released during daylight hours, peaking around midday and at dawn [4], emergence times corresponding to times when their putative hosts are present in the water and available for infection. Given that this is a high schistosomiasis transmission area, it may seem counter-intuitive that very few snails shed cercariae. However, this is not entirely new and our findings are consistent with other studies from endemic areas with high transmission where few or none of the snails collected shed any cercariae. In a very early study by McClelland [22], it was noted that although 90% of school children were infected with *S. haematobium*, there were difficulties in finding infected snails. Elsewhere, in contrast to the high human prevalence of *S. haematobium *infection in Msambweni along the Kenyan coast, the proportion of snails shedding *S. haematobium *cercariae was only 1.2% [18]. Still at the Kenyan coast, another study observed that cercarial shedding was either low (range = 0.14-3.4%) or altogether absent [23]. Previous research along Kisumu beach (one of the sites sampled in the present study) observed that cercarial shedding was lowest during the months of February-April [5]. In addition, the same study observed that the number of snails shedding cercariae at car wash beach (another site sampled in our study) was low (between 0 and 5). In the Lake Victoria basin in western Kenya, only 1.04% (236/22,641) of snails collected at various sites shed cercariae [4], while a recent study in Sesse Islands of Lake Victoria, Uganda, reported that none of the snails collected shed any cercariae [3].

Several explanations may be put forward for the absence or low numbers of snails shedding cercariae. First, it has been suggested that the percentage of infected snails may be very low or cercariae may be shed for only a limited period of time [22]. This confounded with the focal nature of schistosomiasis and the complexity of sampling vast areas where snails may be dispersed makes it difficult to accurately pin-point which site would contain high numbers of infected snails. Second, snail population abundance, infection rates and cercarial output are also under seasonal influence [5,18,24]. Perhaps it may not be optimal for snails to shed cercariae around the peak rainy season of April-May (as occurs in Kisumu), when there may be decreased water contact activities associated with swimming and or domestic use. Third, the very low proportion of infected snails along the lakeshore (0.3%) may also be due to enhanced dilution of human faecal matter associated with mixing across a larger volume of water or perhaps, difficulties in miracidia locating snail hosts in an undulating aquatic environment as has been suggested elsewhere [21]. Fourth, cercarial release from field-collected snails may also be inhibited by a variety of contaminants and invertebrates harbored by the snails. For instance, besides adherence and blockage of the centre whorl of the shells of *Biomphalaria*, bdelloid rotifers are known to emit a small molecular weight component that can cause a reversible paralysis of *S. mansoni *cercariae and limit cercarial release from patent snails [25]. Fifth, it has been suggested that field snails in heavily endemic areas are subjected to pulses of infection rather than to a continuous flow of miracidia [26]. Considering the fact that prepatent infection can last for several weeks with only a proportion of snails reaching the stage of cercarial shedding [27], and that prepatent infection rates can be substantial, and exceed patent infection rates [28], it is also plausible that majority of snails sampled in our study may have had prepatent infections. Clarification of such prepatent infections may be done using methods such as snail crushing in search of larvae or repeated shedding in the laboratory over time, although such methods are unsuitable for accurate and large-scale monitoring. This may be necessary especially in light of the observation that as a method, cercarial emergence (which is routinely used), severely underestimates parasite prevalence [29]. Although it is generally accepted that finding infected snails is the only confirmation of transmission of the disease, our findings suggest that a cautious interpretation of transmission based on snail infection is necessary. Moreover, a single, brief exposure to cercariae-infested water is sufficient to effect transmission [30], even where the number of shedding snails is low [31].

### Implications of snail data in designing cost-effective control interventions

Elucidating snail abundance may have implications in devising cost-effective control interventions. For instance if snail abundance is very high and infection levels among human population is low, mollusciding rather than chemotherapy may be advocated for. In addition, initiation (timing) and frequency of chemotherapy may be planned effectively, depending on the snail densities and re-infection rates at particular sites. Accurate evaluation of the risk of infection will also benefit from information on both snail infection and presence and distribution of cercariae. Changes in the environment associated with man's activities e.g. water impoundment (as was the case with dams in our study), human pollution and over-fishing may also influence the diversity and abundance of freshwater snails which may modify schistosomiasis transmission.

### Limitations of the current study

Several potential limitations are noted in our study. First, snails were sampled on a single day at each site. Given the known local and seasonal variation in snail abundance in the field [5,18], future surveys may be enhanced by sampling snails for at least two consecutive days and at different times of the year for more precise snail abundance determination. Second, in many endemic areas, animal and human schistosomes may appear together in the same transmission sites, necessitating species identification of schistosome cercariae. However, we did not quantify or identify schistosome cercariae species specific to humans. Such identification may largely benefit from developed molecular techniques such as PCR [24]. Similarly, identification of snails in our study was based on morphological characteristics alone. Future studies may benefit from use of more sensitive molecular techniques to verify identity of snails. This would be of importance, for instance considering the current taxonomical problems associated with separation of the two *Biomphalaria *species i.e. *B. pfeifferi *and *B. sudanica *[10,32], and also in the *Bulinus africanus *group (i.e. *B. globosus *and *B. nasutus*) [33].

## Conclusions

In summary, our findings indicate that besides Lake Victoria, active transmission of schistosomiasis occurs from inland habitats within the informal areas of Kisumu City and in streams that flow into the Lake, and suggests that transmission patterns are closely related to the abundance and spatial distribution of vector snails. The location and timing of snail control interventions will not only depend on the spatial distribution of snail densities, but also on their temporal variations. Since schistosomiasis transmission tends to be focal, focal mollusciciding, improvements in local sanitation and hygiene as well as public health awareness, would be advocated to complement chemotherapy in reducing transmission and re-infection in such urban settings.

## Abbreviations

DVBNTD: Division of Vector-Borne and Neglected Tropical Diseases; GIS: geographical information system; GPS: global positioning system; WHO: World Health Organization.

## Competing interests

The authors declare that they have no competing interests.

## Authors' contributions

The study was designed by PNMM, MRO and DMS. Fieldwork, processing and snail identification was undertaken by SO. The data was compiled by SO, and analyzed by SO and MRO. MRO, WGZO, PNMM and DMS provided scientific guidance in data collection, planning and implementation of day-to-day field and laboratory activities. The manuscript was prepared by SO and MRO, all authors actively contributed to the interpretation of the findings. All authors read and approved the final manuscript.
